# Persistent DNA damage triggers activation of the integrated stress response to promote cell survival under nutrient restriction

**DOI:** 10.1186/s12915-020-00771-x

**Published:** 2020-03-30

**Authors:** Elena Clementi, Larissa Inglin, Erin Beebe, Corina Gsell, Zuzana Garajova, Enni Markkanen

**Affiliations:** Institute of Veterinary Pharmacology and Toxicology, Vetsuisse Faculty, University of Zürich, 8057 Zürich, Switzerland

**Keywords:** DNA repair, DNA base-excision repair, DNA base damage, DNA single-strand breaks, DNA double-strand breaks, DNA damage response, Integrated stress response, Tumour microenvironment, Tumour stroma

## Abstract

**Background:**

Base-excision repair (BER) is a central DNA repair mechanism responsible for the maintenance of genome integrity. Accordingly, BER defects have been implicated in cancer, presumably by precipitating cellular transformation through an increase in the occurrence of mutations. Hence, tight adaptation of BER capacity is essential for DNA stability. However, counterintuitive to this, prolonged exposure of cells to pro-inflammatory molecules or DNA-damaging agents causes a BER deficiency by downregulating the central scaffold protein XRCC1. The rationale for this XRCC1 downregulation in response to persistent DNA damage remains enigmatic. Based on our previous findings that XRCC1 downregulation causes wide-ranging anabolic changes, we hypothesised that BER depletion could enhance cellular survival under stress, such as nutrient restriction.

**Results:**

Here, we demonstrate that persistent single-strand breaks (SSBs) caused by XRCC1 downregulation trigger the integrated stress response (ISR) to promote cellular survival under nutrient-restricted conditions. ISR activation depends on DNA damage signalling via ATM, which triggers PERK-mediated eIF2α phosphorylation, increasing translation of the stress-response factor ATF4. Furthermore, we demonstrate that SSBs, induced either through depletion of the transcription factor Sp1, responsible for XRCC1 levels, or through prolonged oxidative stress, trigger ISR-mediated cell survival under nutrient restriction as well. Finally, the ISR pathway can also be initiated by persistent DNA double-strand breaks.

**Conclusions:**

Our results uncover a previously unappreciated connection between persistent DNA damage, caused by a decrease in BER capacity or direct induction of DNA damage, and the ISR pathway that supports cell survival in response to genotoxic stress with implications for tumour biology and beyond.

## Background

DNA damage is considered the molecular origin of many pathophysiological processes such as ageing, neurodevelopmental and neurodegenerative disorders, and cancer [1–3]. Sources of DNA damage include a wide variety of exogenous damaging agents. However, even in the absence of exogenous noxious influences, DNA is prone to spontaneous alterations, due to its chemical reactivity in the aquatic milieu and reactive side-products that are created by the cellular metabolism [4]. This leads to a high level of DNA lesions even under physiological ‘unstressed’ circumstances that are in constant need of repair to avert potential mutagenic and cytotoxic consequences [5]. Base-excision repair (BER) is a centrally important DNA repair mechanism responsible for correcting many of these small base lesions and single-strand breaks [6]. BER is a highly coordinated process in which the scaffold protein XRCC1 occupies a critical role due to its ability to stabilise the two other core BER components DNA polymerase β and DNA ligase III [7, 8]. Therefore, depletion of XRCC1 leads to loss of the core BER machinery, and cells deficient in XRCC1 display reduced DNA repair which causes an accumulation of persistent DNA damage and an increase in genomic instability [6, 9]. DNA repair deficiencies have been strongly implicated in the development of cancer, due to increased mutation rate, interference with transcription, or generation of toxic double-strand breaks (DSBs), all of which can lead to cellular transformation [10–14]. Likewise, haploinsufficiency in XRCC1 enhances formation of precancerous lesions [15], and selective depletion of neural XRCC1 or Ape1, another BER component, leads to the development of brain tumours in mice [16, 17]. Moreover, expression of XRCC1 has been shown to be decreased in various different tumours, with lower XRCC1 levels associated with higher proliferation and shorter overall survival [18–23]. Hence, to avoid deleterious consequences of mutations and maintain DNA integrity, the capacity for DNA repair has to be tightly adapted to cellular needs [24, 25].

Recently, we have demonstrated that prolonged exposure to the pro-inflammatory cytokine TGFβ or reactive oxygen species (ROS) causes a deficiency in BER by decreasing XRCC1 expression in human primary fibroblasts [26]. This decrease in XRCC1 levels resulted in a lowered BER capacity and led to accumulation of persistent DNA damage. Mechanistically, persistent DNA damage was found to induce ATM-dependent degradation of the transcription factor Sp1, which controls XRCC1 expression, therefore creating a BER deficiency through decreasing transcription of XRCC1 [27]. Thus, somewhat counterintuitive to what would be expected for maintenance of DNA integrity in response to stressful conditions, cellular BER capacity is curbed through downregulation of XRCC1 in response to persistent DNA damage. The rationale for this reduction of BER capacity in response to persistent DNA damage remains enigmatic.

We have previously shown that a BER deficiency, brought about by downregulation of XRCC1 by siRNA, induces wide-ranging gene-expression changes in cellular metabolism that are comparable to changes found in tumours [28]. This reprogramming of cellular metabolism led to anabolic changes upon BER deficiency and was found to be at least partially dependent on the stress-responsive transcription factor ATF4 [26, 28]. As key component of the integrated stress response (ISR), ATF4 is a transcription factor that induces metabolic adaptations to stressful conditions to ensure survival [29]. As such, ATF4 mounts appropriate responses in response to a variety of different stresses, including nutrient deprivation, hypoxia, viral infections, and endoplasmic reticulum stress, and it has been shown to have important roles in cancer [30, 31]. Activation of the ATF4 and ISR in response to these stressors is mediated through activation of one of the stress-responsive eIF2α kinases GCN2, PERK, HRI, or PKR, which in turn phosphorylate eIF2α. While phosphorylation of eIF2α leads to a global repression of translation, it selectively increases the translation of ATF4 due to alternate use of open reading frames in the 5′ region of the ATF4 mRNA [30]. ATF4 controls expression of a wide range of genes that allow adaptation of cells to stressful surroundings to promote cell survival, e.g. via induction of autophagy, but—depending on the context—can also induce apoptosis [31].

Based on our observation that XRCC1-depletion caused wide-ranging anabolic changes in cellular metabolism, we hypothesised that the downregulation of BER in response to persistent stressful influences could enhance cellular survival under suboptimal conditions, such as nutrient restriction. Here, we demonstrate that persistent DNA damage, caused by a decrease in BER capacity or direct induction of DNA damage, triggers activation of the ISR pathway through ATM, PERK, peIF2α, and ATF4 to enhance cellular survival under nutrient-restricted conditions. Our results uncover a previously unappreciated connection between the BER capacity, persistent DNA damage signalling, and ISR, constituting a novel mechanism to support cell survival in response to genotoxic stress that has strong implications for tumour biology and beyond.

## Results

### XRCC1 KD imparts human fibroblasts with a survival advantage in nutrient-restricted conditions

**Fig. 1:**
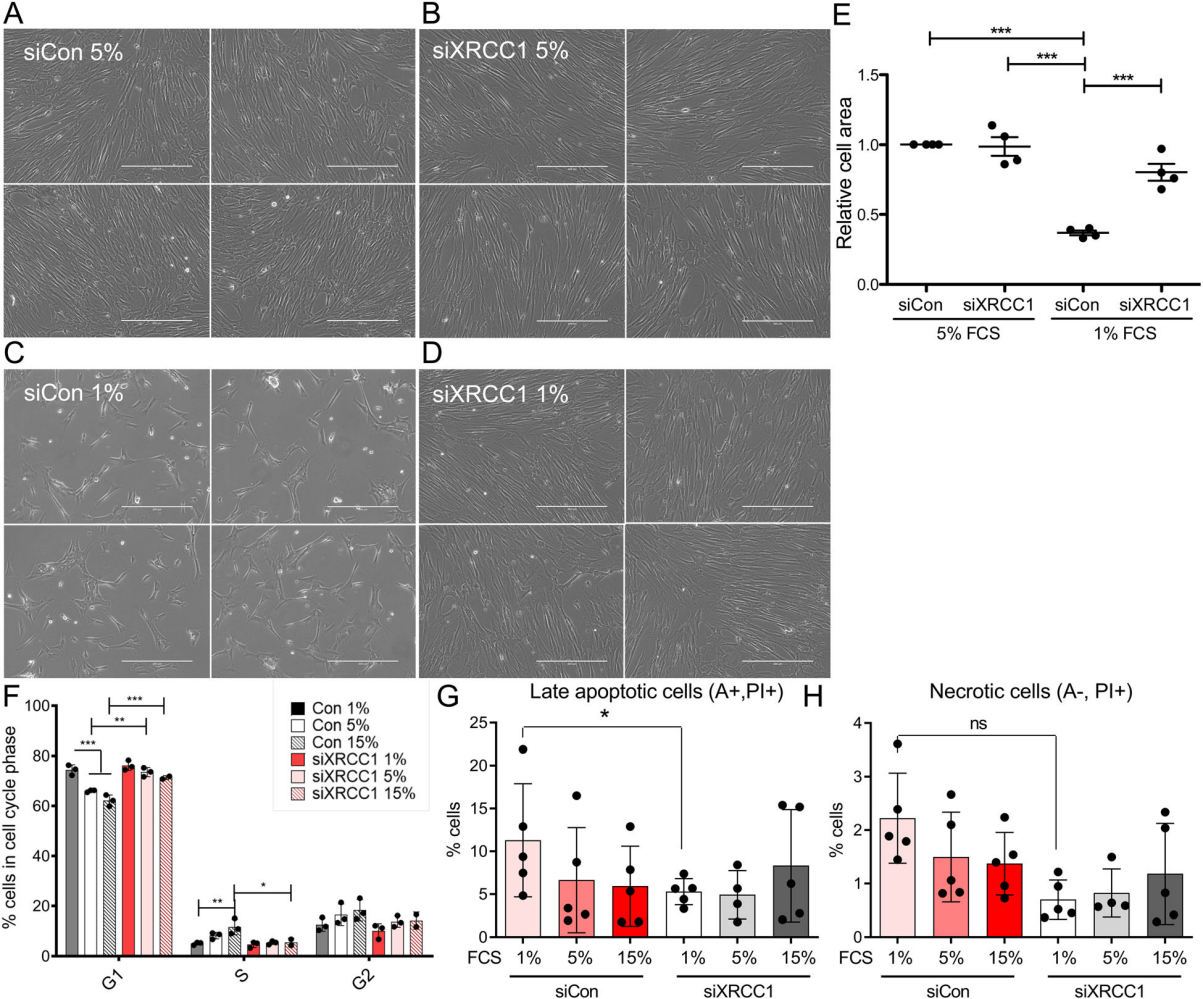
Selective survival advantage of XRCC1 KD cells in nutrient-restricted conditions. Phase-contrast images of Tig-1 cells treated with siControl (**a**, **c**) or siXRCC1 (**b**, **d**) and grown in a medium containing 5% FCS (**a**, **b**) or 1% FCS (**c**, **d**). Images are from one representative experiment, with four different fields randomly chosen per condition. Scale bar = 400 μm. **e** Quantification of relative cell area as shown in **a**–**d**, normalised to siControl grown at 5% FCS. *n* = 4. Only statistically significant differences are indicated using asterisks. **f** Cell cycle analysis of siControl and siXRCC1 cells cultured at 15%, 5%, and 1% FCS, respectively. Shown is the percentage of cells in the respective cell cycle phases. *n* = 3. Statistically significant differences are indicated using asterisks. **g** Quantification of the percentage of cells in late apoptosis in siControl and siXRCC1 cells cultured at 15%, 5%, and 1% FCS, respectively. *n* = 3. *p < 0.05. **h** Quantification of the percentage of necrotic cells in siControl and siXRCC1 cells cultured at 15%, 5%, and 1% FCS, respectively. *n* = 3. n.s. not significant

Why do persistently stressed cells lower the expression of XRCC1? Based on our previous findings, we hypothesised that the downregulation of BER capacity would lead to a metabolic rewiring towards nutritional self-sufficiency that would be advantageous for cells to survive under nutrient-starved conditions. To test this hypothesis, Tig-1 human primary fibroblasts were treated with XRCC1 or control siRNA and reseeded into dishes with medium containing varying amounts of foetal calf serum (FCS) 24 h later. Cell morphology and density were analysed 72 h after knockdown (KD) using phase-contrast images. When grown in a medium containing 15 or 5% FCS, no obvious differences in cell number or morphology were discernible between XRCC1 KD and control cells (Fig. 1a, b, e, and Additional file 1, Figure S1A and B). Interestingly, however, when FCS was lowered to 1%, XRCC1 KD cells clearly displayed a significantly higher cell number, a more elongated fibroblast-like cell morphology, and less rounded and floating cells compared to controls (Fig. 1c, d, e). KD efficiency and transcriptional response of known genes upregulated in response to XRCC1 KD (ACTA2, PALLD [26]) were not affected by reseeding cells into the different FCS conditions (Additional file 1, Figure S1C). These results were validated using two additional siRNA sequences against XRCC1 (Additional file 2, Figure S2) and in two other human primary fibroblast cell lines (Additional files 3 and 4, Figures S3 and S4). The increased cell number of XRCC1 KD cells under low FCS conditions could be due to either an increase in proliferation, a decrease in apoptosis, or a combination of both. To differentiate between these possibilities, we analysed the cell cycle distribution of control and XRCC1 KD cells grown in 1%, 5%, and 15% FCS, respectively, using flow cytometry. As expected from previous findings [28], XRCC1 KD cells grown at 15% FCS displayed a significant increase in G1 cells and a concomitant decrease in S-phase cells compared to controls, suggesting that XRCC1 KD under these conditions causes the cell cycle to slow down slightly by prolonging the G1-phase, presumably due to the accumulation of persistent DNA damage (Fig. 1f). Similarly to this, cell cycle distribution at 5% still displayed significantly more XRCC1 KD cells in G1 and slightly less in S-phase compared to control cells. However, in cells grown at 1% FCS, no differences in the percentage of cells in G1, S, or G2 cell cycle phases could be detected between control and XRCC1 KD cells. Indeed, the amount of control cells in G1 significantly increased and cells in S-phase significantly decreased with lower FCS concentrations, while the percentage of XRCC1 KD cells in the different cell cycle phases remained stable throughout the different FCS conditions. Thus, XRCC1 KD cells did not have a proliferative advantage over control cells at low FCS conditions. To understand whether apoptosis differed between control and XRCC1 KD cells grown in low FCS, we analysed the rate of apoptotic cells by Annexin V/propidium iodide staining at 72 h after seeding into different FCS-containing media. While no significant changes between control and siXRCC1 cells at 15% and 5% FCS could be detected, there were significantly more late apoptotic control cells than siXRCC1 cells when cultured at 1% (Fig. 1g). A similar trend was observed with respect to necrotic cells, but this difference did not reach statistical significance (Fig. 1h). Of note, these values are likely to strongly underestimate the real amount of dying cells in the control situation, since most of the dead cells are already lost before the analysis (Fig. 1c, d). The observed increase in apoptotic cell death of control cells is also in accordance with the phenotype, as the control clearly shows much more rounded, detaching, and floating cells than the XRCC1 KD (Fig. 1c, d). In summary, these results strongly support the hypothesis that XRCC1 KD confers cells with a survival advantage in nutrient-restricted conditions.

### XRCC1 KD induces the integrated stress response through PERK-peIF2α-ATF4 signalling to support cell survival in nutrient-restricted conditions

**Fig. 2:**
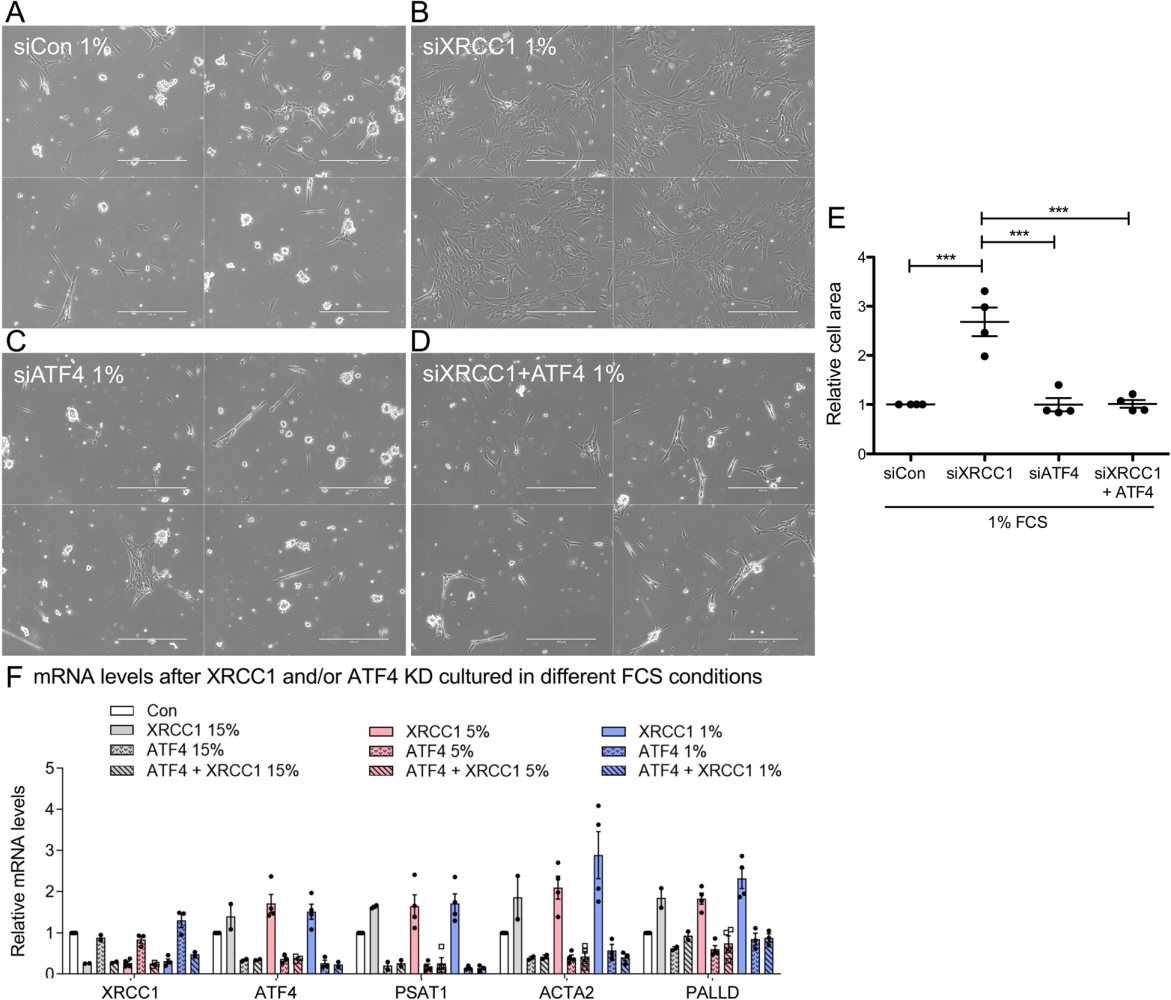
Selective survival advantage of XRCC1 KD cells under nutrient restriction depends on ATF4. Phase-contrast images of Tig-1 cells treated with siControl (**a**), siXRCC1 (**b**), siATF4 (**c**), or siXRCC1 and siATF4 (**d**) and grown in a medium containing 1% FCS. Images are from one representative experiment, with four different fields randomly chosen per condition. Scale bar = 400 μm. **e** Quantification of relative cell area as shown in **a**–**d**, normalised to siControl grown at 1% FCS. *n* = 4. Only statistically significant differences are indicated using asterisks. **f** Relative mRNA levels of XRCC1, ATF4, PSAT1, ACTA2, and PALLD after siXRCC1 in cells cultured at 15, 5, and 1% FCS, respectively, normalised to expression in the respective control cells. *n* = 4

BER depletion in fibroblasts through KD of XRCC1 has been shown to induce transcription of ATF4 by a yet unknown mechanism [26, 28]. ATF4 is a transcription factor that can induce metabolic adaptations to stressful conditions and thereby promote survival [29–31]. Therefore, we asked whether the survival advantage of fibroblasts in nutrient-restricted conditions upon XRCC1 KD depended on ATF4. At 5% FCS, depletion of ATF4 alone or in combination with XRCC1 merely showed a slight decrease in cell numbers compared to control cells, suggesting that a baseline ATF4 level is required for optimal cellular fitness (Additional file 5, Figure S5A-D). Strikingly, when cultured at 1% FCS, combined KD of ATF4 and XRCC1 completely rescued the phenotype observed with XRCC1 KD alone and was virtually indistinguishable from control cells (Fig. 2a–e). KD efficiency was not influenced by FCS conditions, and functionality of the ATF4 KD was further validated by a strong decrease in expression of PSAT1, a known direct downstream target of ATF4, as well as rescue of ACTA2 and PALLD expression, which are known to be upregulated after XRCC1 KD through an ATF4-dependent mechanism (Fig. 2f) [26]. It is important to note that these XRCC1 KD-induced changes on both transcript and protein levels could be observed irrespective of the FCS conditions, but the phenotype of increased survival over control cells only manifested upon FCS restriction (Fig. 2f and data not shown). We conclude that increased cell survival upon XRCC1 KD under nutrient-restricted conditions depends on the transcription factor ATF4.

**Fig. 3:**
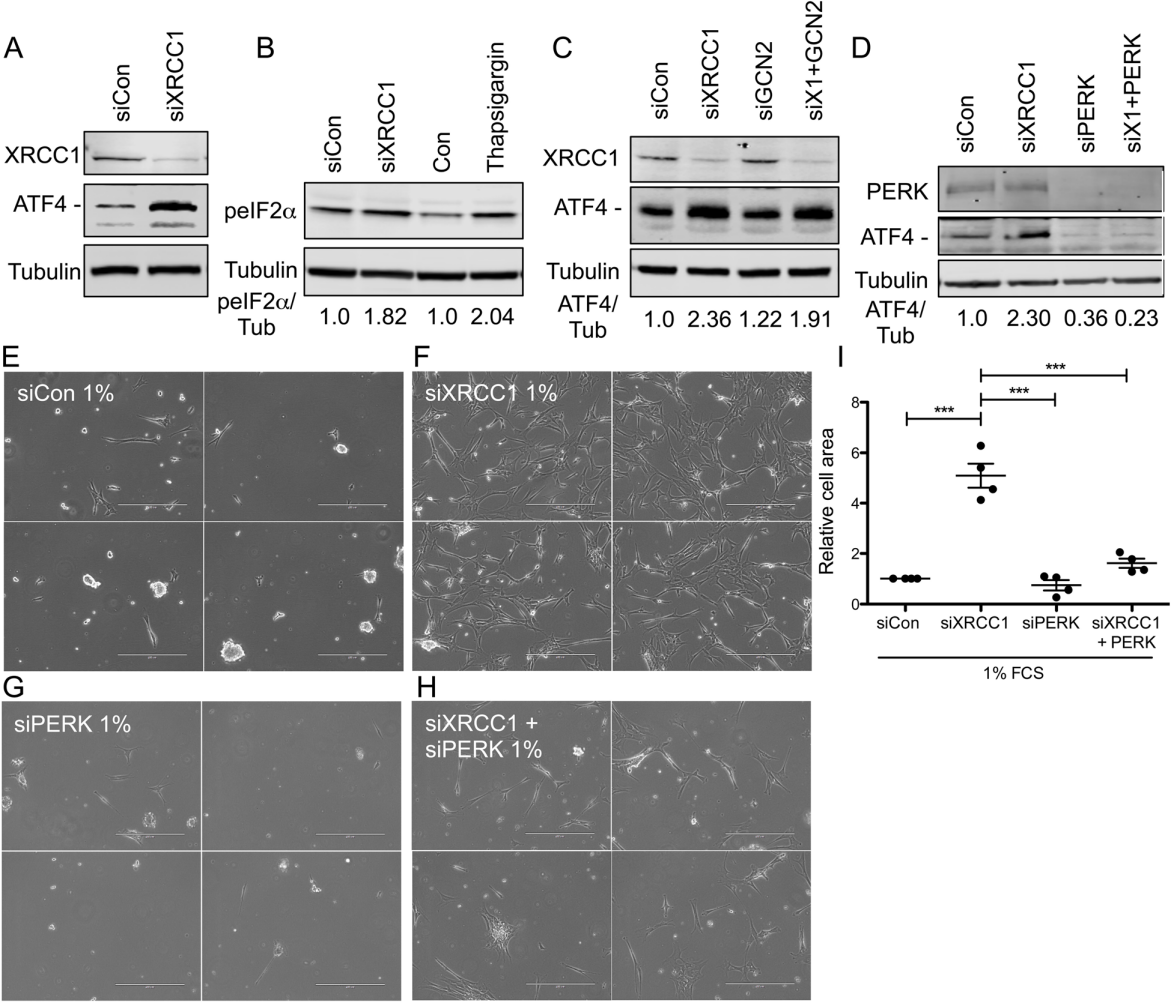
XRCC1 KD induces the integrated stress response through PERK to support survival under nutrient-restricted conditions. **a**–**d** Representative Western blots of **a** ATF4 and XRCC1 levels after siXRCC1. The band specific for ATF4 is indicated with a dash. *n* = 3. **b** peIF2α after siXRCC1 or Thapsigargin. Normalised peIF2α levels are shown below the lanes. *n* = 3. **c** ATF4 and XRCC1 after KD of XRCC1 and/or GCN2. The band specific for ATF4 is indicated with a dash. Normalised levels of ATF4 are shown below the lanes. *n* = 3. **d** PERK and ATF4 after KD of XRCC1 and/or PERK. The band specific for ATF4 is indicated with a dash. Normalised levels of ATF4 are shown below the lanes. *n* = 3. **e**–**h** Phase-contrast images of cells treated with **e** siControl, **f** siXRCC1, **g** siPERK, or **h** siXRCC1 and siPERK, and grown in medium containing 1% FCS. Images are from one representative experiment with four different fields randomly chosen per condition. Scale bar = 400 μm. **i** Quantification of relative cell area as shown in **e**–**h**, normalised to siControl grown at 1% FCS. *n* = 4. Only statistically significant differences are indicated using asterisks

As a central effector of the ISR, ATF4 can be regulated on the translational level by activation of the ISR [30]. To determine whether XRCC1 KD resulted in an increase in ATF4 protein levels, we analysed ATF4 levels in XRCC1 KD cells. Western blot analysis revealed a strong increase of ATF4 protein upon XRCC1 KD (Fig. 3a), which was further validated using two other siRNA sequences against XRCC1 (Additional file 5, Figures S5E and F). We next wondered whether XRCC1 KD could induce phosphorylation of eIF2α (peIF2α) and thereby increase ATF4 translation through activation of the ISR. In line with an increase in ATF4 translation through activation of the ISR by XRCC1 KD, Western blot analysis confirmed that XRCC1 KD induced peIF2α (Fig. 3b). Of note, the extent of peIF2α activation by XRCC1 KD was comparable with its induction by the known ER-stress inducer Thapsigargin. These results prompted the question, which of the upstream kinases was responsible for XRCC1 KD-mediated activation of the ISR that led to upregulation of ATF4. GCN2 and PERK have been shown to be important in response to amino acid and glucose starvation as well as endoplasmic reticulum stress [32]. While ATF4 upregulation upon XRCC1 KD was not altered by co-KD of GCN2 (Fig. 3c and Additional file 5, Figure S5G), co-KD of PERK completely abrogated ATF4 induction (Fig. 3d). Importantly, co-KD of PERK with XRCC1 also annulled the increased survival of XRCC1 KD cells at 1% FCS (Fig. 3e–i). Similar results were obtained by inhibition of PERK using GSK2606414 in combination with XRCC1 KD (Additional file 6, Supplementary Figure S6) [33]. Hence, these findings suggest that XRCC1 KD-mediated activation of the ISR proceeds through the PERK-peIF2α-ATF4 signalling pathway, which manifests in a survival advantage of cells under nutrient restriction.

### The XRCC1 KD-induced survival advantage in nutrient-restricted conditions is induced through signalling of persistent DNA single-strand breaks via ATM

**Fig. 4:**
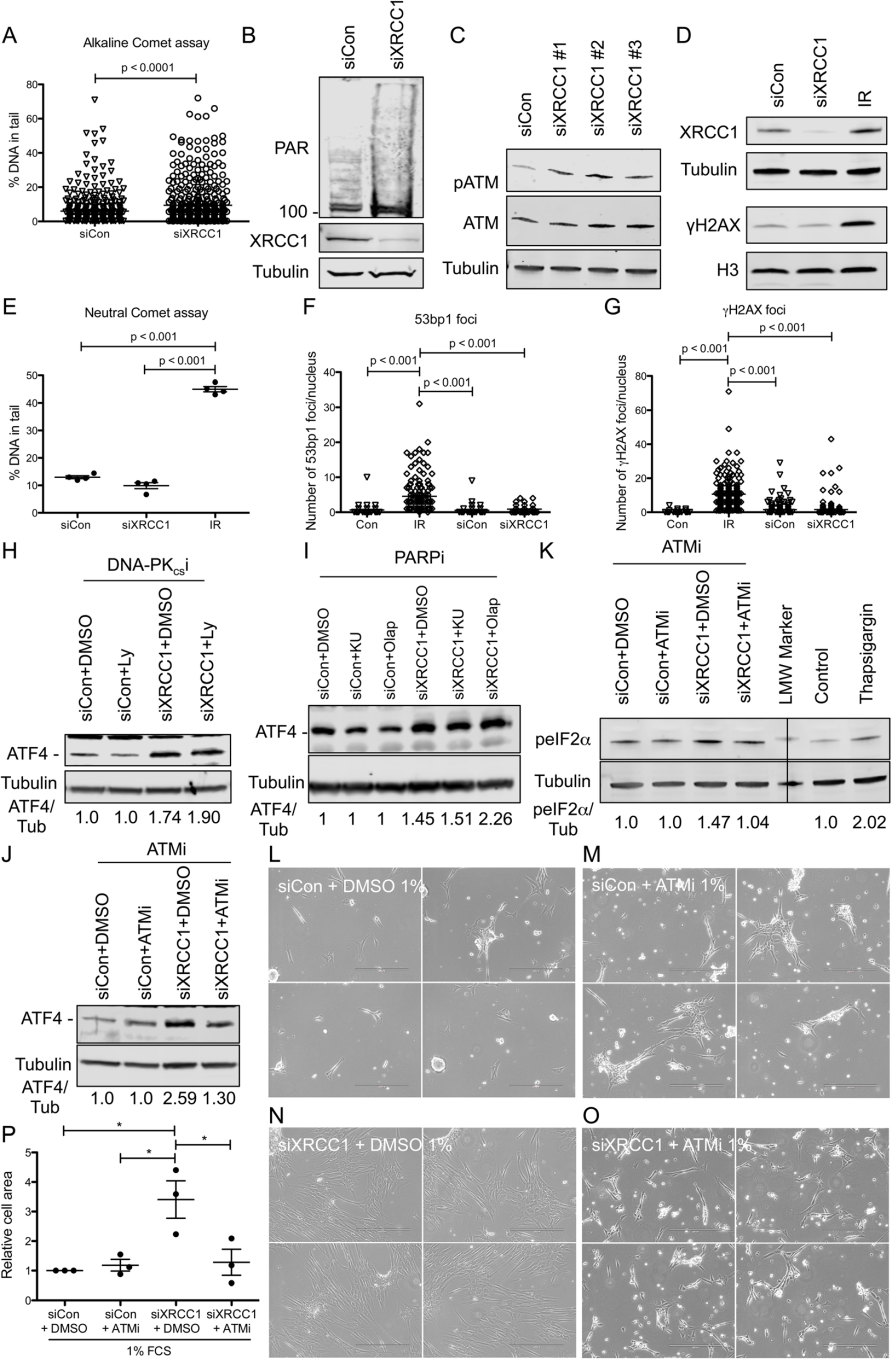
The XRCC1 KD-induced activation of the ISR is mediated through signalling of persistent single-stranded DNA damage via ATM. **a** Alkaline comet assay of siControl and siXRCC1 cells. **b**–**d** Representative Western blots of **b** PAR and XRCC1 after siXRCC1. The smear above 100 kDa marker indicates PAR. **c** pATM and ATM after siXRCC1 using 3 different siRNA sequences. **d** γH2AX, Histone H3, XRCC1, and Tubulin in cells after siXRCC1, or 4 Gy IR. **e** Neutral comet assay of cells treated after siControl, siXRCC1, or 4 Gy IR. *n* = 4. **f**, **g** Quantification of 53 bp1 (**f**) or γH2AX (**g**) foci per nucleus in cells after 4 Gy IR, siControl, or siXRCC1. **h**–**j** Representative Western blots of **h** ATF4 in siControl or siXRCC1 cells treated with Ly or DMSO; **i** ATF4 in siControl or siXRCC1 cells treated with KU, Olap, or DMSO; **j** ATF4 in siControl or siXRCC1 cells treated with ATMi or DMSO; and **k** peIF2a in siControl or siXRCC1 cells treated with ATMi or DMSO. Normalised levels of ATF4 or peIF2α are shown below the lanes. **l**–**o** Phase-contrast images of siControl (**l**, **m**) or siXRCC1 (**n**, **o**) cells treated with DMSO (**l**, **n**) or ATMi (**m**, **o**), grown in a medium containing 1% FCS. Images are from one representative experiment, with four different fields randomly chosen per condition. Scale bar = 400 μm. **p** Quantification of relative cell area as shown in **l**–**o** normalised to siControl + DMSO grown at 1% FCS. *n* = 3. For all quantifications, only statistically significant differences are indicated using asterisks

Given the connection between depletion of XRCC1 and the survival advantage in nutrient-restricted conditions mediated through activation of the ISR, we sought to identify the mechanism by which XRCC1 KD could induce the ISR. It is well established that XRCC1 KD induces persistent DNA damage due to BER deficiency, which leads to poly-ADP-ribose (PAR) formation [7, 8, 25, 26, 28, 34, 35]. In line with this, XRCC1 KD cells displayed an increase in persistent DNA damage as measured by the alkaline comet assay (Fig. 4a), increased PAR formation (Fig. 4b), and phosphorylation of ATM (Fig. 4c). Importantly, consistent with previous reports [35], XRCC1 KD Tig-1 cells did not accumulate DSBs as evidenced by a lack of increase in γH2AX by Western blot (Fig. 4d), no increase in DSBs as assessed by the neutral comet assay (Fig. 4e), and no change in the number of 53bp1 or γH2AX foci in the cell nuclei (Fig. 4f, g). Therefore, we asked whether DNA damage signalling was involved in the activation of the ISR upon XRCC1 KD. SSBs and DNA base damage have been shown to induce signalling through PARP, ATM, and DNA PK_cs_ [35–37]. Neither inhibition of DNA PK_cs_ nor PARP using two different inhibitors could rescue the increase in ATF4 expression upon XRCC1 KD (Fig. 4h, i, and Additional file 7, Figure S7A). In contrast, inhibition of ATM was able to rescue the induction of ATF4 (Fig. 4j and Additional file 7, Figure S7B). ATM inhibition also rescued the increase in peIF2α upon XRCC1 KD (Fig. 4k). Importantly, while not affecting growth at 5% FCS, ATM inhibition completely rescued the increased survival imparted by XRCC1 KD at 1% FCS (Fig. 4l–p and Additional file 7, Figure S7C). Thus, increased survival in nutrient-restricted conditions through activation of the ISR by XRCC1 KD depends on signalling of SSBs by ATM.

### Manipulation of the XRCC1 transcription factor Sp1, single-strand breaks induced by low-level H_2_O_2_, and DNA double-strand breaks induced by ionizing radiation also activate the ISR-mediated cell survival under nutrient-restricted conditions

**Fig. 5:**
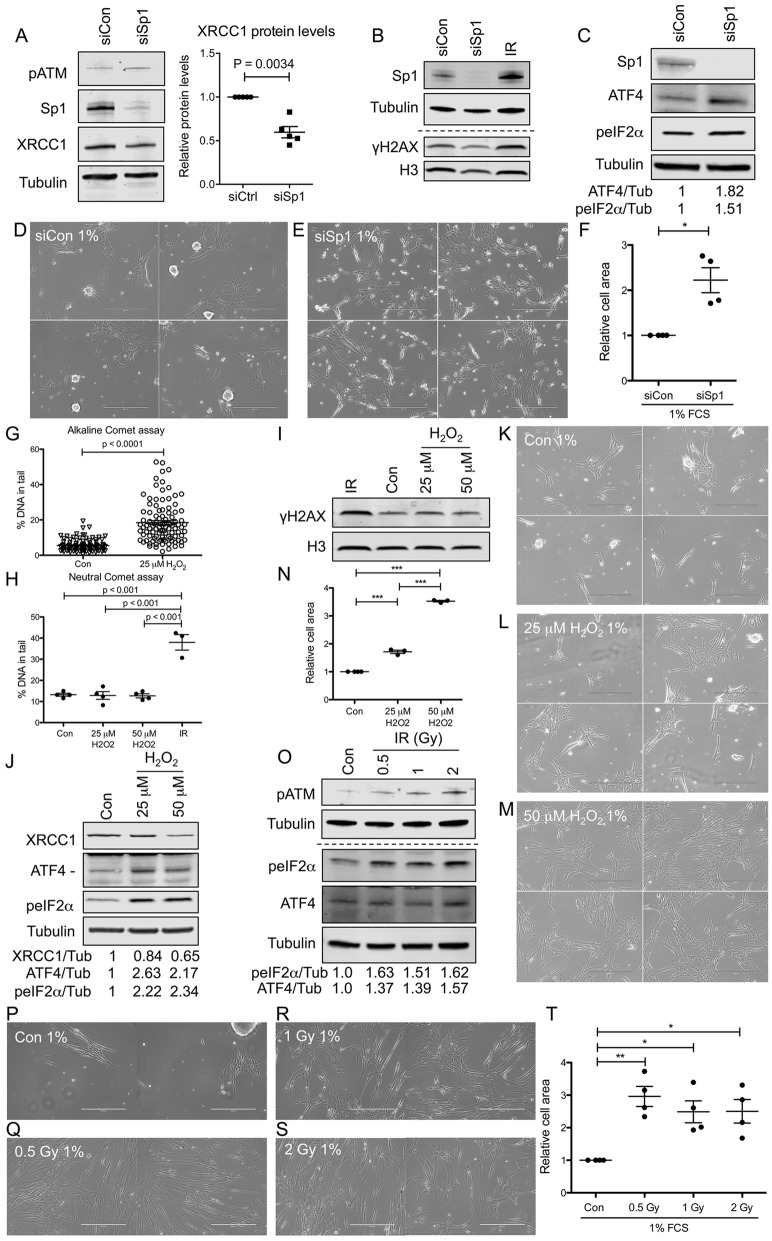
Induction of ISR-mediated cell survival under nutrient-restricted conditions through KD of Sp1, direct SSBs induced by H_2_O_2_, or direct DSBs induced by ionising radiation. **a** (left) Representative Western blot of pATM, Sp1, and XRCC1 levels after siSp1. (right) Quantification of XRCC1 levels shown on the left, *n* = 5. **b**, **c** Representative Western blot of **b** Sp1, Tubulin, γH2AX, and Histone H3, or **c** Sp1, ATF4, and peIF2α after siSp1. Normalised levels of ATF4 and peIF2α are indicated. **d**–**e** Phase-contrast images of cells treated with siCon (**d**) or siSp1 (**e**), grown in a medium containing 1% FCS. **f** Quantification of **d** and **e**, *n* = 4. **g** Alkaline comet assay of cells treated with 25 μM H_2_O_2_. **h** Neutral comet assay of cells pre-treated with 25 μM or 50 μM H_2_O_2_ for three consecutive days, or 4 Gy IR 15 min prior to harvesting, respectively. *n* = 4. **i**, **j** Representative Western blots of **i** γH2AX and Histone H3 or **j** XRCC1, ATF4, and peIF2α, in cells treated as in **h**. Normalised levels of XRCC1, ATF4, and peIF2α are indicated. **k**–**m** Phase-contrast images of cells treated as in **h** prior to seeding into a medium containing 1% FCS. **n** Quantification of **k**–**m**. *n* = 4. **o** Western blot of pATM, Tubulin, ATF4, and peIF2α in control cells or cells treated with 0.5, 1, or 2 Gy of IR on three consecutive days. Normalised levels of ATF4 and peIF2α are indicated. **p**–**s** Phase-contrast images of control cells (**p**) or cells treated as in **o** prior to seeding into a medium containing 1% FCS. **t** Quantification of **p**–**s**, *n* = 4

We then wondered whether activation of the ISR could also be triggered by other modalities that lead to a decrease in XRCC1 levels. Expression of XRCC1 is controlled by the transcription factor Sp1, and depletion of Sp1 leads to a deficiency in BER due to decreased transcription of XRCC1 [27]. Consistent with this, KD of Sp1 for 72 h led to a significant reduction of XRCC1 protein levels, as well as an increase in pATM, supporting the notion that Sp1 KD leads to persistent DNA damage due to a depletion in XRCC1 protein levels (Fig. 5a). Of note, Sp1 KD did not elevate γH2AX levels (Fig. 5b). Accordingly, Sp1 KD also triggered an increase in both ATF4 and peIF2α levels (Fig. 5c), suggesting that the transcriptional downregulation of XRCC1 of approximately 40% (Fig. 5a) obtained by KD of Sp1 is sufficient to trigger activation of the ISR. In line with this, Sp1 KD cells displayed significantly increased survival compared to siControl-treated cells when cultured at 1% FCS, but not at 5% FCS (Fig. 5d–f, Additional file 8, Figure S8A and B).

Next, we asked if the observed phenomenon could also be elicited by direct induction of persistent DNA damage. For this, we analysed ATF4 and peIF2α levels in cells exposed to low levels of hydrogen peroxide (H_2_O_2_) for 72 h, which caused a slight increase of SSBs compared to control cells (Fig. 5g), but failed to induce DSBs as assessed by the neutral comet (Fig. 5h) and γH2AX levels (Fig. 5i). Exposure of cells to 25 or 50 μM H_2_O_2_ over 72 h led to an upregulation of ATF4 and peIF2α protein, in accordance with an induction of the ISR by persistent DNA damage (Fig. 5j). Of note, this treatment also precipitated a decrease in XRCC1 protein levels (Fig. 5j). Strikingly, such chronic exposure to low levels of H_2_O_2_ before seeding into low FCS medium induced a significant survival advantage in these cells compared to control treated cells upon culture at 1% FCS (Fig. 5k–n). Hence, persistent single-strand DNA damage brought about by chronic exposure to low levels of H_2_O_2_ induces an ISR-dependent selective survival advantage under nutrient-restricted conditions.

Finally, since ATM is well-known to be activated upon DSB formation by ionising radiation [38], we wondered whether its activation by direct formation of DSBs could also induce the ISR. Indeed, Tig-1 cells irradiated for three consecutive days with doses of 0.5, 1, or 2 Gy, respectively, displayed elevated levels of pATM as well as peIF2α and ATF4 (Fig. 5o). Importantly, cells pre-treated in this way with ionising radiation before reseeding also displayed increased survival upon culture in 1% FCS compared to control cells (Fig. 5p–t). From these results, we conclude that activation of ATM through DSBs also leads to induction of the ISR to support cell survival under nutrient restriction.

Collectively, our data clearly demonstrate that persistent DNA damage, as induced via downregulation of XRCC1 or direct induction of SSBs or DSBs through DNA-damaging agents, confers cells with a selective survival advantage in nutrient-restricted conditions through an ATM-dependent activation of the ISR pathway involving the PERK-peIF2α-ATF4 axis. As such, these results uncover a previously unappreciated connection between the BER capacity, DNA damage signalling, and the ISR, which supports cell survival in response to genotoxic stress with strong implications for tumour biology and other physiological conditions in which cells have been shown to selectively decrease the levels of BER components.

## Discussion

As a mainstay of genome integrity, BER is a centrally important pathway to counteract DNA lesions that arise due to constant exposure of living organisms to exogenous and endogenous DNA-damaging agents. Hence, tight adaptation of BER capacity to cellular needs is considered critically important, as both insufficient or uncontrolled repair activity could potentially lead to the accumulation of DNA damage and mutations [6, 24, 25, 39]. Under most physiological circumstances, BER capacity is sufficient to avoid accumulation of unrepaired DNA damage. Acute genotoxic stress could lead to temporal exhaustion of BER capacity, leading to delays in repair of DNA damage, potentially giving rise to genetic instability. Importantly, however, there are also numerous physiological circumstances in which cells have been described to selectively induce a BER deficiency [40–44]. While the general idea has been that downregulation of BER in these cells might ‘prime’ them for apoptosis or removal by the immune system, evidence for this is still inconclusive. Indeed, several aspects suggest a physiological role for this transient attenuation of BER capacity that goes beyond simple priming of cells for apoptosis and removal, as outlined in the following. Circulating monocytes have been shown to harbour a deficiency in DNA repair due to downregulation of key repair proteins [40, 45, 46]. However, dendritic cells or macrophages that are derived from these monocytic precursors re-express the relevant repair proteins, becoming repair-proficient again. It would quite simply not make much sense to try and initially target all of these precursor cells by default for apoptosis or removal by the immune system as a step during their maturation even before they have reached their final differentiation state. Similarly, chronic hypoxia has been found to lead to a decrease in expression of several BER proteins, and human spermatozoa harbour a ‘truncated BER pathway’ manifested by the absence of XRCC1 and Ape1 [44]. Finally, human primary fibroblasts that are exposed to prolonged treatments with pro-inflammatory cytokines or oxidative stress strongly decrease their BER capacity due to a downregulation of XRCC1 and other BER proteins [25–27]. Importantly, human fibroblasts are considered highly apoptosis-resistant, and to undergo senescence much rather than apoptosis, and they can be maintained in culture for extremely long periods in that state [47]. This is in line with the crucial role of fibroblasts to maintain the organism’s structural integrity, because, even in a post-replicative senescent state, fibroblasts are still able to actively shape the extracellular matrix. This would not be possible where damaged fibroblasts are quickly eliminated through apoptosis or by the immune system. Following up on the idea that the specific downregulation of BER capacity could serve a physiological purpose, our work uncovers a previously unappreciated connection between the BER, persistent DNA damage, and the ISR that supports cell survival in response to genotoxic stress. These findings have strong implications for tumour biology in a variety of aspects. On the one hand, these results could directly impact on cells in the tumour microenvironment, the so-called cancer-associated stroma, and especially cancer-associated fibroblasts (CAFs), which are highly central players in the development and progression of tumours [48, 49]. Here, persistent DNA damage signalling could increase survival and self-sufficiency of stromal tumour-supportive cells. Interestingly, ROS has been shown to be a potent inducer of CAFs [50]. In line with this, we have shown that persistent DNA damage in fibroblasts leads to a secretory phenotype by which fibroblasts are able to support growth and metastatic ability of tumour cells [26]. Thus, we propose that under stressful circumstances, such as protracted inflammation or persistent ROS, DNA damage is induced in the tissue-resident fibroblasts, which leads to ATM signalling that initiates ATF4-dependent metabolic reprogramming to increase cellular self-sufficiency in nutrients, thereby enabling these cells to survive even in nutrient-starved conditions. Through this increased resilience, fibroblasts can still fulfil their important structural roles in the organism, as they are the main responsible for synthesis of the extracellular matrix, that provides the structural scaffold for other cells to grow on and mechanical properties that determine the integrity of organs [51]. Such a resilience mechanism seems important especially also for CAFs. *Per definitionem*, CAFs are located closely adjacent to the epithelial tumour-forming, rapidly dividing cancer cells. These cancer cells have a huge demand in a wide variety of nutrients to sustain their high proliferative rate, and they have been shown to remodel the surrounding stroma in a way that increases the delivery of such nutrients and other growth-promoting molecules [48]. Specifically, CAFs have been found to take up metabolic waste of cancer cells to produce metabolites that in turn are secreted again to feed the tumour [52]. It is thus thought that most of the ‘rich’ nutrients are drained away from CAFs to support tumour growth, which results in the necessity for CAFs to survive in a ‘starved’ environment. The observed downregulation of BER capacity in fibroblasts exposed to a maintained pro-inflammatory stimulation that induces CAF generation seems to thus increase self-sufficiency of these cells, which would be beneficial in a starved environment. In summary, our observations suggest the metabolic rewiring upon BER decline after stressful insults to be a physiological response of fibroblasts to ensure their survival in nutrient-starved conditions, in order to fulfil their principal role of maintaining the structure of the organism. On the other hand, our findings could also have implications for survival of pre-malignant or malignant tumour cells, which are strongly exposed to various different types of stress [31]. However, the reaction of tumour cells to persistent DNA damage with regard to activation of the ISR and their survival under nutrient starvation remains to be addressed in the future.

Mechanistically, we found activation of the ISR by XRCC1 depletion to depend on signalling of SSBs via ATM, which activates PERK-mediated eIF2α phosphorylation and increases translation of the transcription factor ATF4 (Figs. 3 and 4). Currently, it remains unclear whether activation of PERK by ATM is direct or indirect. Additionally, also other modalities that induced SSBs (Sp1 KD and treatment with low levels of H_2_O_2_) and even direct induction of DSBs using ionising radiation led to the activation of the ISR-mediated survival phenotype (Fig. 5). Interestingly, cell death that is observed under acute exposure of control cells to nutrient-starved conditions very much resembles the ‘foamy cell death’ that has recently been described in response to chronic ER stress [53]. Prevention of this form of cell death also depends on PERK activation of peIF2α, similarly to what we observe upon XRCC1 KD. Additionally, this foamy cell death apparently seems to be driven by a ‘non-apoptotic’ mechanism, which would explain why the quantification of apoptotic cells through Annexin V-PI staining does fully recapitulate the extent of cell death that is visible under nutrient-deprived conditions in control cells (Fig. 1). Indeed, it seems that the Annexin V-PI staining only captures a very small proportion of the effect that acute nutrient starvation has on the control cells. It will be interesting to determine what exact mechanism precipitates cell death under these conditions.

The transcription factor ATF4 has a dual role in cells promoting either their adaptation to endure stress or the induction of apoptosis [31]. The ATF4 dependence of the metabolic reprogramming that we observed upon both XRCC1 KD and induction of DSBs is nicely in line with our previous findings demonstrating increased ATF4 transcription after XRCC1 KD [28] as well as its involvement in the transcription of several CAF markers that are activated upon XRCC1 KD [26]. Hence, cells with persistent DNA damage seem somehow to be able to exploit ATF4, to reduce stress resulting from nutrient limitation and benefit from its pro-survival effects. This raises intriguing questions as to what exact ATF4-dependent changes allow cells to thrive in nutrient-starved conditions and why ATF4 promotes cellular survival after persistent DNA damage under these conditions rather than inducing apoptosis. A deeper understanding of these mechanisms might also facilitate the identification of pharmacological strategies to fine-tune ATF4 activity in different pathologies, such as cancer.

## Conclusion

Our results uncover a previously unappreciated connection between persistent DNA damage, caused either by a decrease in BER capacity or by direct induction of DNA single-strand or double-strand breaks, and activation of the ISR. Activation of the ISR by DNA damage relies on phosphorylation of the DNA damage sensor protein ATM, which leads to PERK-mediated eIF2α phosphorylation, increasing translation of the stress-response factor ATF4. This mechanism supports cell survival in response to genotoxic stress with strong implications for tumour biology and beyond.

## Methods

### Cell culture

TIG-1, AG09319, and AG16409 primary human fibroblasts were purchased from Coriell and cultured under standard conditions (37 °C, 5% CO_2_) in Gibco™ DMEM, low glucose, GlutaMAX™ Supplement, pyruvate containing 15% foetal calf serum (FCS). For all treatments using inhibitors, inhibitors were dissolved in DMSO, medium was changed, and fresh inhibitor was added every 24 h. The following inhibitors were used: PERKi GSK2606414 (MerckMillipore), used at 1 μM final concentration; DNA-PK_cs_i LY294002 (Ly, MerckMillipore), used at 1 μM final concentration; ATMi Ku-60019 (ATMi, SigmaAldrich), used at 10 μM final concentration; and PARPi Ku-0058948 (Ku; AxonMedChem) and Olaparib (Olap; Selleck Chemicals), both used at 1 μM final concentration. As positive control to induce ER stress/peIF2α/ATF4, cells were exposed for 3 h to 1 μM Thapsigargin (Sigma). To induce DNA double-strand breaks, cells were irradiated using a Faxitron Cabinet X-ray system Model RX-650 at the indicated IR doses.

### siRNA transfection

Transfections with siRNA purchased from Eurogentec or Thermo Fisher were carried out using Lipofectamine RNAiMAX reagent (Invitrogen) according to the manufacturer’s specifications. Cells were analysed at indicated time points after transfection. siRNA sequences used are listed in Additional file 9, Table S1.

### Analysis of cell growth in serum-restricted growth conditions

10^6^ cells were seeded into 10-cm dishes 24 h prior to siRNA transfection in standard medium containing 15% FCS. Twenty-four hours after transfection, cells were washed and trypsinised with 1 ml trypsin solution, and trypsin was neutralised through addition of 2 ml of DMEM containing 5% FCS. One ml of the cell solution was distributed into each fresh 10-cm dish, containing 10 ml of medium with different concentrations of FCS (i.e. 15, 5, and 1%), and incubated another 72 h until analysis. Images of random fields were obtained with a phase-contrast microscope at ×10 magnification, and cells were collected for further analysis. For experiments involving pre-treatment of cells with H_2_O_2_ or IR, cells were grown in 10-cm dishes in standard medium containing 15% FCS for the duration of the pre-treatment. Pre-treatment was performed thrice every 24 h using the indicated doses of H_2_O_2_ or IR. For treatments with H_2_O_2_, the medium was changed every time. Twenty-four hours after the third round of pre-treatment, equal numbers of cells were seeded into different FCS-containing dishes as described above. To quantify the relative cell area from phase-contrast images, the area covered with cells was quantified after thresholding of images to remove the background using ImageJ software. For each experiment and data point, two to four independent fields were quantified, and mean values were calculated, which were then normalised to the respective control. Every experiment was independently repeated three to four times (*n* = 3 or 4), as indicated in the respective figure legends. Data are expressed as individual data points and mean ± SEM. Raw data can be found in Additional file 12 Table S4.

### qRT-PCR

Total RNA was purified using the RNeasy® Mini Kit by QIAGEN according to the manufacturer’s protocol. Equal amounts of RNA were reverse transcribed using the BioRad iScript™ cDNA Synthesis Kit according to the manufacturer’s protocol with the LabCycler (SensoQuest). Quantitative real-time PCR (RT-qPCR) was performed using the KAPA SYBR® FAST One-Step qRT-PCR Kit in a total volume of 10 μl in duplicates on the CFX384 Touch™ Real-Time PCR detection system (Bio-Rad). The comparative CT method was applied for quantification of gene expression, values were normalised against GAPDH and B2M and the control, and results were expressed as fold change in mRNA levels over control cells. Each experiment was independently repeated between two and four times as indicated in the figure legends, and data are expressed as individual data points and mean ± SD. Primers are detailed in Additional file 10 [54], Table S2, and were ordered from Microsynth. Raw data can be found in Additional file 12, Table S4.

### Flow cytometry

For cell cycle analysis by FACS, trypsinised cells were fixed in ice-cold 70% ethanol for at least 30 min at − 20 °C. To remove the fixation solution, cells were spun 5 min at 250 rcf at 4 °C, and the supernatant was discarded. Cells were then resuspended in phosphate-buffered saline with 100 μg/ml of DNase free RNase A (Sigma) and incubated at 37 °C for 30 min and further stained with 10 μg/ml propidium iodide (Sigma). Samples were run on a Fortessa (BD Biosciences) and the cell cycle distribution analysed using FlowJo V10.6.1. Each experiment was independently repeated three times. Data are mean ± SD of *n* = 3 independent experiments and are expressed as individual data points and mean ± SD. Analysis of apoptotic and necrotic cells was performed with the Annexin V-FITC Apoptosis Staining/Detection Kit (Abcam, ab14085) according to the manufacturer’s protocol. Briefly, cells treated as indicated were washed and adherent cells trypsinised. Trypsin was neutralised using serum containing medium, and 500,000 cells were collected by centrifugation and resuspended in 500 μl 1X Binding Buffer. Five μl of Annexin V-FITC and 5 μl propidium iodide were added, and samples were incubated at room temperature for 5 min before acquisition with FACS as detailed above. Each experiment was independently repeated five times. Data are mean ± SD of *n* = 5 independent experiments and are expressed as individual data points and mean ± SD. Raw data can be found in Additional file 12 Table S4.

### Western blot

For Western blotting, unless otherwise stated, whole cell extracts that were prepared from cells grown in 15% FCS were used according to a procedure described previously [28]. 20 to 40 μg of total protein extract was separated on 4–20% Tris–Glycine gels (Novex) and transferred onto Immobilon-FL polyvinylidene fluoride (PVDF) membranes (Millipore) according to standard procedures (Novex). Blots were probed with primary antibodies detailed in Additional file 11 Table S3, and secondary antibodies conjugated with Alexa Fluor 680 and IRDye 800CW (both Li-cor Biosciences). Detection and quantification was performed using the Odyssey CLX image analysis system (Li-cor Biosciences). Tubulin or Histone H3 serves as the loading control. Each experiment was independently repeated three to five times. For quantification, protein levels were first normalised to the loading control and then to the respective control lane. Relative protein levels are indicated below the lanes and refer to the blot that is pictured.

### Alkaline comet assay

The alkaline comet assay was performed as described [8]. Briefly, cells were harvested by trypsinisation, diluted to a concentration of 2 × 10^5^ cells/ml in medium, and embedded on a microscope slide in 1% low-melting agarose in phosphate-buffered saline (PBS) (Bio-Rad) that was settled on ice. Slides were lysed in buffer containing 2.5 M NaCl, 100 mM ethylenediamine-tetraacetic acid (EDTA), 10 mM Tris–HCl pH 10.5, 1% (v/v) dimethyl sulfoxide (DMSO), and 1% (v/v) Triton X-100 for 1 h at 4 °C. Slides were then incubated in the dark for 30 min in cold electrophoresis buffer (300 mM NaOH, 1 mM EDTA, 1% (v/v) DMSO, pH > 13) to allow DNA unwinding prior to electrophoresis at 21 V for 25 min in the comet assay tank from Trevigen. Neutralisation of the slides was performed with 0.5 M Tris–HCl (pH 8.0). The slides were stained with SYBR Gold (Invitrogen) and analysed using the Open Comet plugin for Fiji [55]. To quantify, a minimum of 50 cells were analysed per assay and condition, and the assay was repeated independently three to four (Fig. 4) times. All individual data points from all repeats and their mean ± SD are displayed.

### Neutral comet assay

The neutral comet assay was performed as described in [56]. Briefly, cell harvesting and embedding was performed as described for the alkaline comet assay. As positive control, cells were irradiated with 4 Gy IR 15 min prior to harvesting. Slides were lysed in buffer containing 2.5 M NaCl, 100 mM EDTA, 10 mM Tris, and 1% *N*-laroylsarcosine, pH 9.5, with freshly added 1% (v/v) DMSO and 0.5% (v/v) Triton X-100 for 1 h at 4 °C in the dark. Slides were then washed 3 times in electrophoresis buffer (300 mM sodium acetate, 100 mM Tris–HCl, pH 8.3) and incubated for 1 h in fresh electrophoresis buffer. Electrophoresis was performed in fresh cold buffer for 1 h at 21 V (ca 120 mA) in the comet assay tank from Trevigen. Afterwards, slides were rinsed 3 times with dH_2_O, immediately stained using SYBR Gold (Invitrogen), and air dried. Analysis was performed using the Open Comet plugin for Fiji. For each data point, at least 50 cells were quantified per assay and condition, and mean values were calculated. The experiment was independently repeated 3–4 times. Data are mean ± SD of the mean values from these independent experiments, as indicated in the respective figure legends. Raw data can be found in Additional file 12, Table S4.

### Immunofluorescence

Cells were grown in 6-well plates on sterile 12-mm glass coverslips and fixed with 4% paraformaldehyde (pH 8.0) in PBS for 15 min at room temperature. After one wash in PBS, permeabilisation was performed using 0.2% Triton X-100 in PBS for 5 min at room temperature, followed by three washes in PBS. Primary and secondary antibodies were diluted in DMEM with 10% FCS (53 bp1, rabbit, Abcam, 1:1000; γH2AX, mouse, Millipore, 1:1000; anti-mouse Alexa Fluor 488, Thermo Scientific, 1:400; anti-rabbit Alexa Fluor 594, Thermo Scientific, 1:400) and incubated 1–2 h at room temperature. DNA was stained using 4′,6-diamidino-2-phenylindole dihydrochloride (DAPI, 0.2 μg/ml). After 3 washes in PBS, coverslips were mounted on glass slides using ProLong Gold Antifade Mountant (Molecular Probes) and imaged. Automated detection and quantification of foci in nuclei was performed using Fiji. The number of foci was quantified in a minimum of 100 nuclei per condition, with 2 independent repeats. All individual data points from all repeats and mean ± SD are displayed.

### Experimental design and statistical analysis

The exact sample size (*n*) for each experiment is indicated in the respective figure legends. All statistical analysis, calculation, and graphical display were performed with the programme GraphPad Prism (www.graphpad.com). Statistical testing of differences from 3 groups or more was performed using one-way ANOVA followed by Bonferroni’s multiple comparison test, while Student’s *t* test was applied when only two groups were compared. Significance levels are *p < 0.05, **p < 0.01, and ***p < 0.001.

## Supplementary information

**Additional file 1: Figure S1.** No influence of XRCC1 KD in cells grown at 15% FCS.

**Additional file 2: Figure S2.** Selective growth advantage of XRCC1 KD cells at 1% FCS.

**Additional file 3: Figure S3.** Selective growth advantage of AG09319 cells after XRCC1 KD at low FCS.

**Additional file 4: Figure S4.** Selective growth advantage of AG16409 cells after XRCC1 KD at low FCS.

**Additional file 5: Figure S5.** Influence of ATF4 on siXRCC1 phenotype.

**Additional file 6: Figure S6.** Selective growth advantage of XRCC1 KD cells at 1% FCS depends on PERK activity.

**Additional file 7: Figure S7.** Validation of inhibitor activity.

**Additional file 8: Figure S4.** No influence of XRCC1 KD in siSp1 or IR treated cells grown at 5% FCS.

**Additional file 9: Table S1.** siRNA sequences used in this study.

**Additional file 10: Table S2.** List of primers used for qRT-PCR.

**Additional file 11: Table S3.** List of primary antibodies used.

**Additional file 12: Table S4:** All raw data for this study.

## Data Availability

All data generated or analysed during this study are included in the published article and its supplementary data files (Figs. 1, 2, 3, 4, and 5 and Additional files 1, 2, 3, 4, 5, 6, 7, 8, 9, 10, 11, and 12).
