# Supplementary material for: Persistent DNA damage triggers activation of the integrated stress response to promote cell survival under nutrient restriction
Source: BMC Biol. 2020 Mar 30;18:36. doi: 10.1186/s12915-020-00771-x (PMC7106853; doi:10.1186/s12915-020-00771-x)

Clementi *et al,* 2020

Additional Figure S1: A and B) No influence of XRCC1 KD in cells grown at 15% FCS. Phase-contrast images of cells treated with siRNA against Control (A) or XRCC1 (B), grown in medium containing 15% FCS. Images are from one representative experiment (from a total of n = 3 independent experiments), with four different fields randomly chosen on each plate shown per condition. Scale bar = 400 µm. C) Culture in different FCS conditions does not influence KD efficiency. Relative mRNA levels of XRCC1, ACTA2 and PALLD in siControl and siXRCC1 treated cells that were cultured at 15, 5, and 1% FCS, respectively, normalized to expression in the respective control cells. Data are mean ±SD of 4 independent experiments.

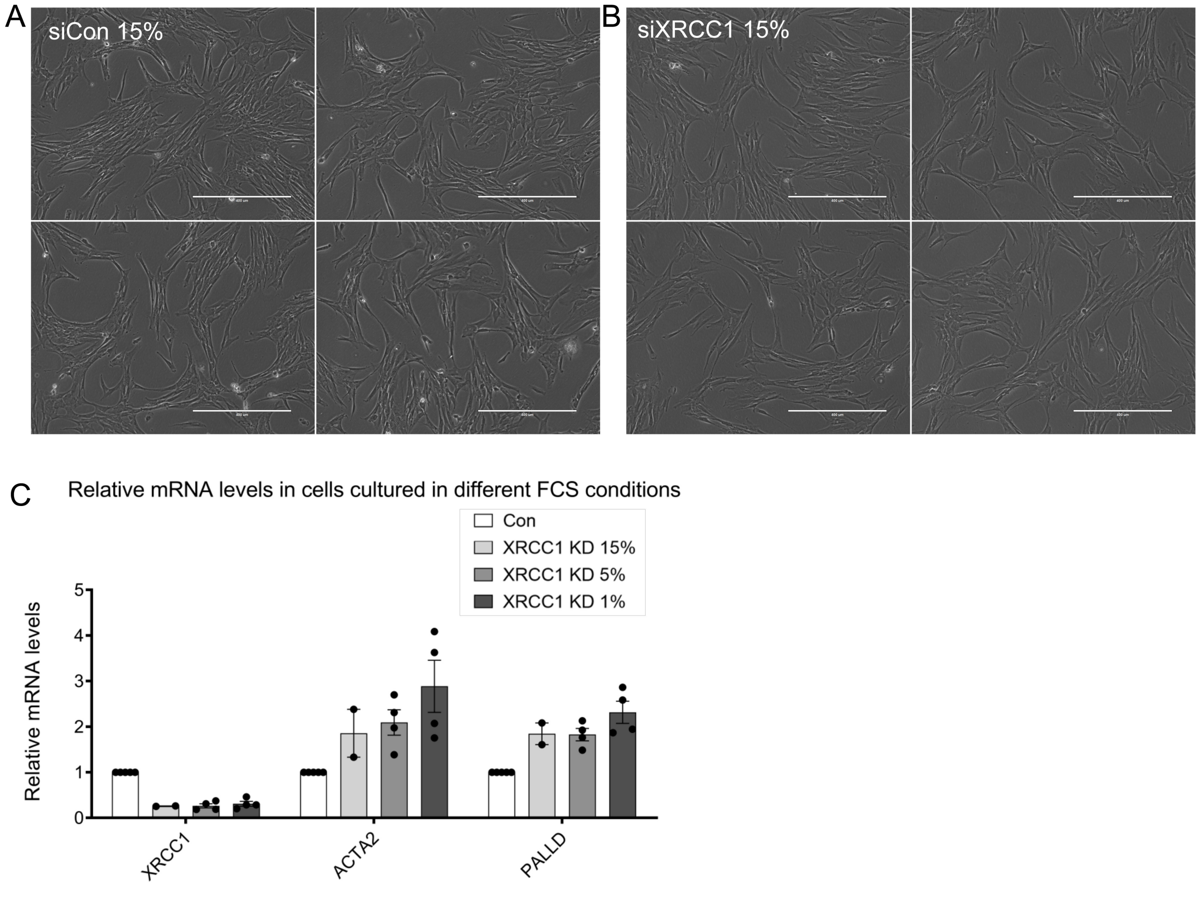

Supplement: Supplementary file 1 — Additional file 1: Figure S1. No influence of XRCC1 KD in cells grown at 15% FCS. [file 12915_2020_771_MOESM1_ESM.pdf]
