# Supplementary material for: Persistent DNA damage triggers activation of the integrated stress response to promote cell survival under nutrient restriction
Source: BMC Biol. 2020 Mar 30;18:36. doi: 10.1186/s12915-020-00771-x (PMC7106853; doi:10.1186/s12915-020-00771-x)

Clementi *et al,* 2020

Additional Figure S2: Selective growth advantage of XRCC1 KD cells at 1% FCS. Phase-contrast images of cells treated with Control siRNA (A and E), XRCC1 siRNA sequence #1 (B and F), XRCC1 siRNA sequence #2 (C and G), or XRCC1 siRNA sequence #3 (D and H) and grown in medium containing 5% FCS (A-D) or 1% FCS (E-H). Images are from one representative experiment (from a total of n = 3 independent experiments). Scale bar = 400 μm.

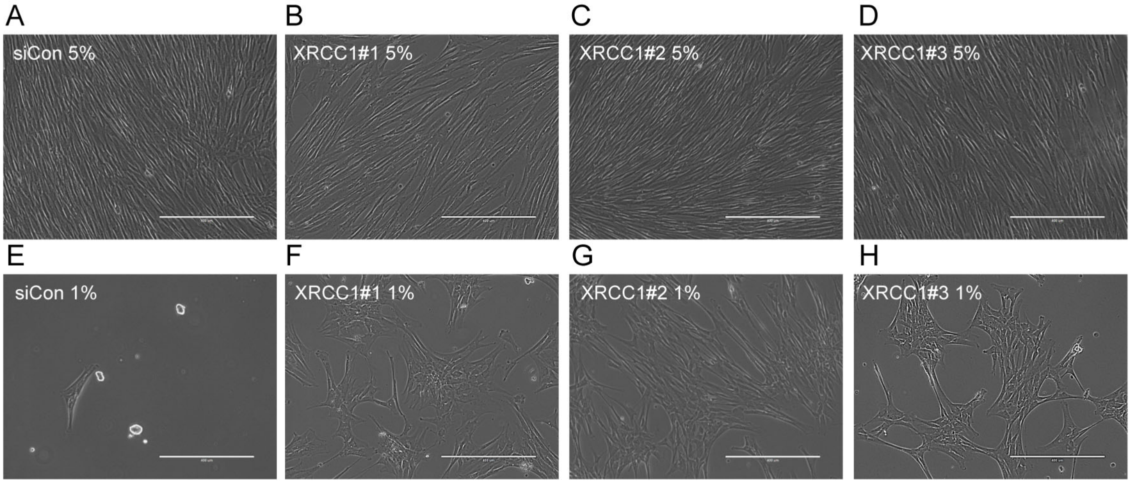

Supplement: Supplementary file 2 — Additional file 2: Figure S2. Selective growth advantage of XRCC1 KD cells at 1% FCS. [file 12915_2020_771_MOESM2_ESM.pdf]
