# Supplementary material for: Persistent DNA damage triggers activation of the integrated stress response to promote cell survival under nutrient restriction
Source: BMC Biol. 2020 Mar 30;18:36. doi: 10.1186/s12915-020-00771-x (PMC7106853; doi:10.1186/s12915-020-00771-x)

Clementi *et al,* 2020

Additional Figure S4: Selective growth advantage of AG16409 cells after XRCC1 KD at low FCS. Phase-contrast images of cells treated with Control (A, C) or XRCC1 siRNA (B, D), and grown in medium containing 5% FCS (A, B) or 2.5% FCS (C, D). Images are from one representative experiment (from a total of n = 3 independent experiments), with four different fields randomly chosen on each plate shown per condition. Scale bar = 400 µm.

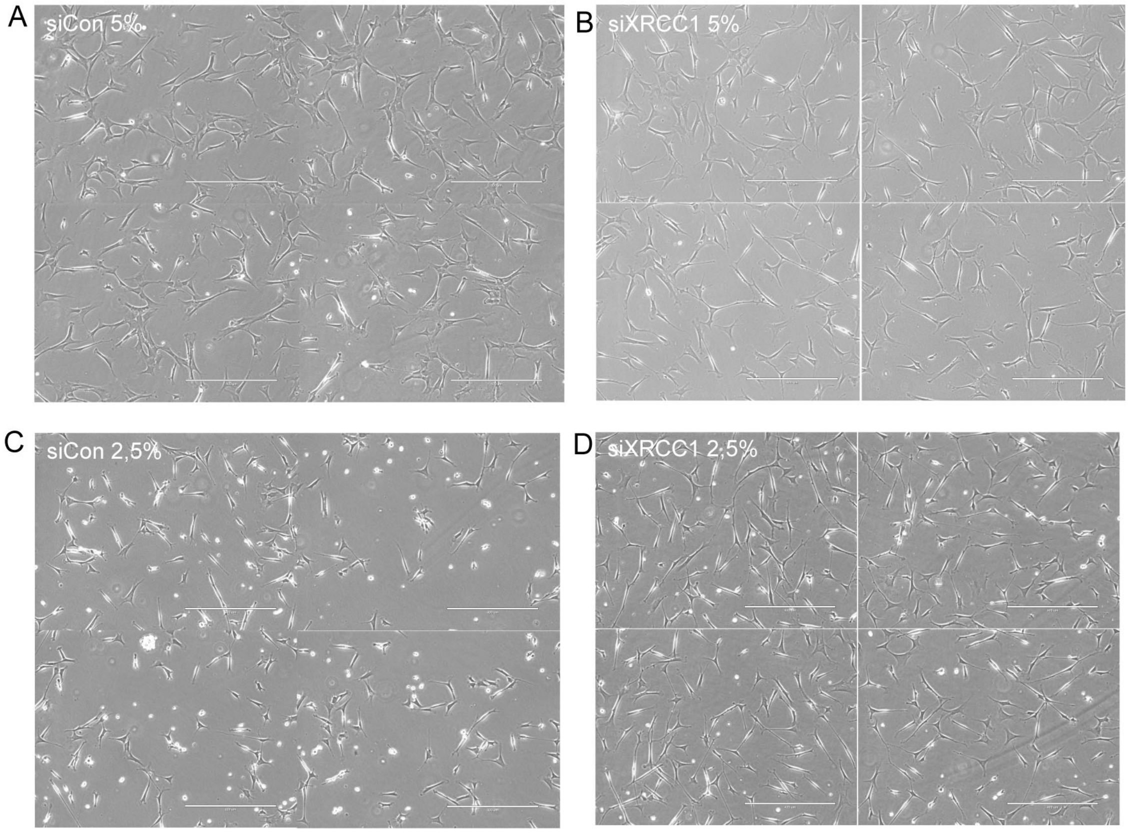

Supplement: Supplementary file 4 — Additional file 4: Figure S4. Selective growth advantage of AG16409 cells after XRCC1 KD at low FCS. [file 12915_2020_771_MOESM4_ESM.pdf]
