# Supplementary material for: Persistent DNA damage triggers activation of the integrated stress response to promote cell survival under nutrient restriction
Source: BMC Biol. 2020 Mar 30;18:36. doi: 10.1186/s12915-020-00771-x (PMC7106853; doi:10.1186/s12915-020-00771-x)
Supplement: Supplementary file 5 — Additional file 5: Figure S5. Influence of ATF4 on siXRCC1 phenotype. [file 12915_2020_771_MOESM5_ESM.pdf]

Additional Figure S5: Influence of ATF4 on siXRCC1 phenotype. A-D) Only subtle influence of XRCC1 and/or ATF4 KD in cells grown at 5% FCS. Phase-contrast images of cells treated with siRNA against A) Control, B) XRCC1, C) ATF4, or D) XRCC1 and ATF4, grown in medium containing 5% FCS. Images are from one representative experiment (from a total of n = 3 independent experiments), with four different fields randomly chosen on each plate shown per condition. Scale bar = 400 µm. E) Western blot analysis of ATF4 upon knockdown of XRCC1 using 3 different siRNA sequences compared to siControl. The band specific for ATF4 is indicated with a dash. Tubulin serves as a loading control. F) Validation of the specificity of the antibody signal for ATF4. The indicated band ATF4 is strongly increased by siXRCC1, and disappears upon KD of ATF4, while the upper, unspecific band is still present after KD. G) Validation of the siRNA knockdown of GCN2. Relative mRNA levels of XRCC1 and GCN2 in cells treated with siControl, siXRCC1, siGCN2, or siXRCC1 and siGCN2, respectively, normalized to expression in the respective control cells. Data are mean ±SD of 2 independent experiments.

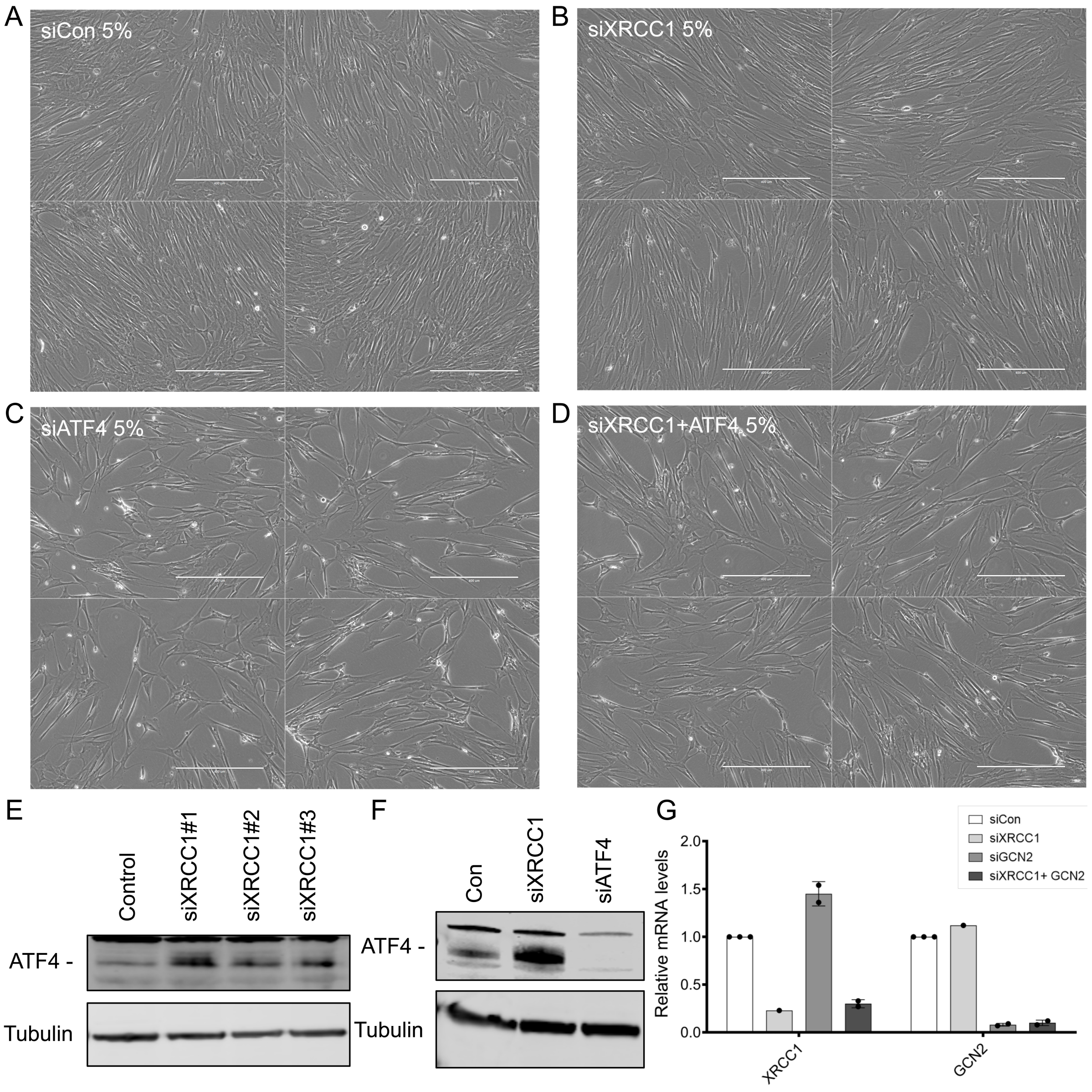
A
siCon 5%
B
siXRCC1 5%
C
siATF4 5%
D
siXRCC1+ATF4 5%
E
Control
siXRCC1#1
siXRCC1#2
siXRCC1#3
ATF4 -
Tubulin
F
Con
siXRCC1
siATF4
ATF4 -
Tubulin
G
siCon
siXRCC1
siGCN2
siXRCC1+ GCN2
Relative mRNA levels
2.0
1.5
1.0
0.5
0.0
XRCC1
GCN2
