# Supplementary material for: Persistent DNA damage triggers activation of the integrated stress response to promote cell survival under nutrient restriction
Source: BMC Biol. 2020 Mar 30;18:36. doi: 10.1186/s12915-020-00771-x (PMC7106853; doi:10.1186/s12915-020-00771-x)
Supplement: Supplementary file 6 — Additional file 6: Figure S6. Selective growth advantage of XRCC1 KD cells at 1% FCS depends on PERK activity. [file 12915_2020_771_MOESM6_ESM.pdf]

Additional Figure S6: Selective growth advantage of XRCC1 KD cells at 1% FCS depends on PERK activity. A-D) Phase-contrast images of Control (A and B) or XRCC1 KD (C and D) cells, treated with DMSO (A and C), or PERK inhibitor GSK2606414 (PERKi) (B and D) and grown in medium containing 1% FCS. Images are from one representative experiment (from a total of n = 3 independent experiments), with four different fields randomly chosen on each plate shown per condition. Scale bar = 400 µm. E-H) Phase-contrast images of Control (E and F) or XRCC1 KD (G and H) cells, treated with DMSO (E and G), or PERK inhibitor (PERKi) (F and H) and grown in medium containing 5% FCS. Images are from one representative experiment (from a total of n = 3 independent experiments), with four different fields randomly chosen on each plate shown per condition. Scale bar = 400 µm. I) Western blot analysis of ATF4 levels after siXRCC1 and/or PERK inhibition (PERKi). The band specific for ATF4 is indicated with a dash. Tubulin serves as loading control. Quantification of ATF4 normalized to tubulin and the respective control are shown below the lanes. K) Western blot analysis of PERK and XRCC1 levels after siXRCC1 and/or PERK inhibition. Note the increased sharpness of the PERK band after PERKi treatment. Tubulin serves as loading control.

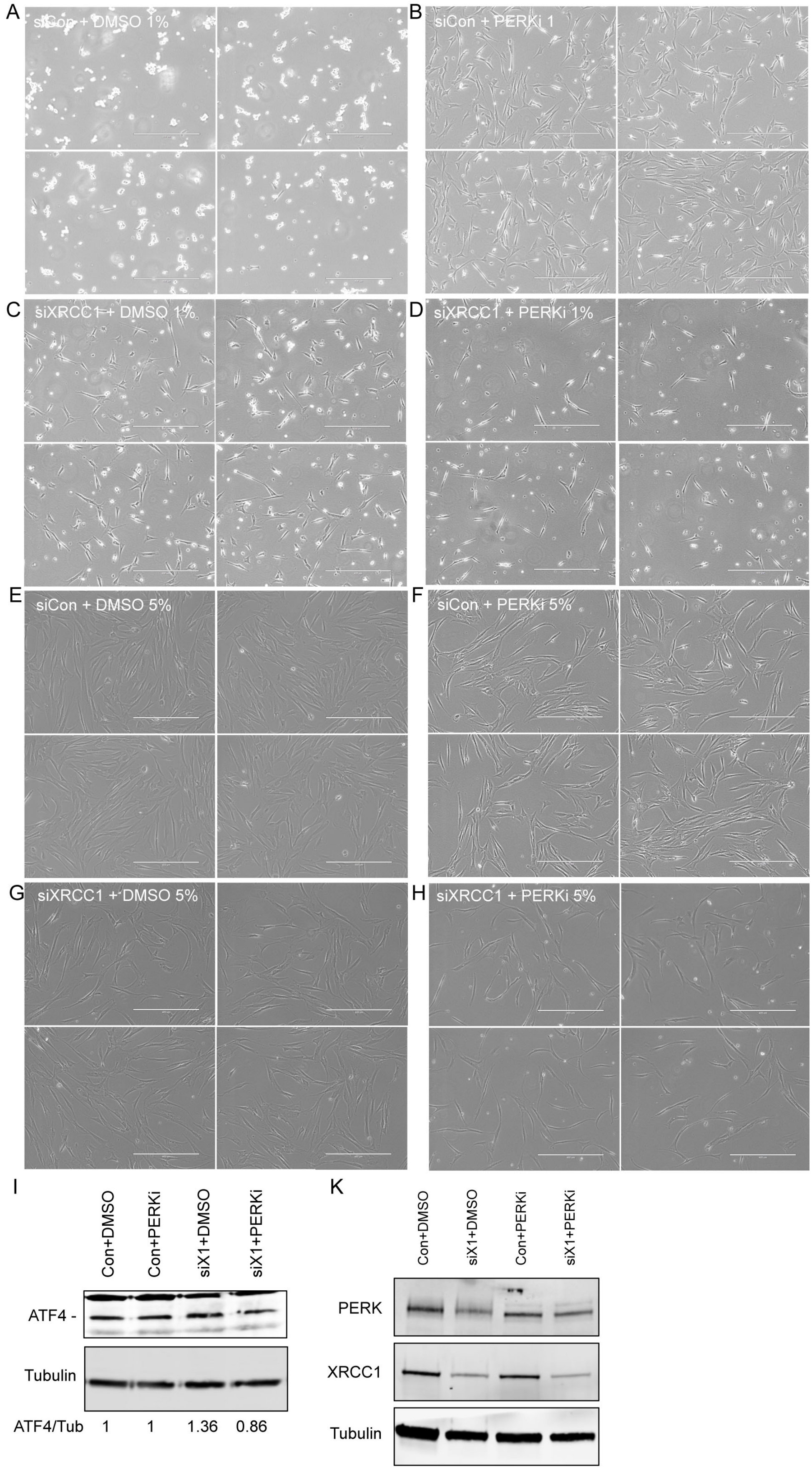
A
siCon + DMSO 1%
B
siCon + PERKi 1
C
siXRCC1 + DMSO 1%
D
siXRCC1 + PERKi 1%
E
siCon + DMSO 5%
F
siCon + PERKi 5%
G
siXRCC1 + DMSO 5%
H
siXRCC1 + PERKi 5%
I
Con+DMSO
Con+PERKi
siX1+DMSO
siX1+PERKi
ATF4 -
Tubulin
ATF4/Tub 1 1 1.36 0.86
K
Con+DMSO
siX1+DMSO
Con+PERKi
siX1+PERKi
PERK
XRCC1
Tubulin
