# Supplementary material for: Persistent DNA damage triggers activation of the integrated stress response to promote cell survival under nutrient restriction
Source: BMC Biol. 2020 Mar 30;18:36. doi: 10.1186/s12915-020-00771-x (PMC7106853; doi:10.1186/s12915-020-00771-x)

Clementi *et al,* 2020

Additional Figure S7: Validation of inhibitor activity. A) Validation of PARP inhibitor treatment by immunofluorescence analysis of PAR formation after a 10 min challenge with 500 μM H2O2 (right) of the cells that were pre-treated with DMSO, KU, or Olaparib for 72 h. Dapi was used to stain nuclei. B) Validation of ATM inhibition by Western blot analysis of p-ATM after a 1 h challenge with 125 uM H2O2 of the cells that were pre-treated with ATMi für 72 h. Tubulin serves as loading control.

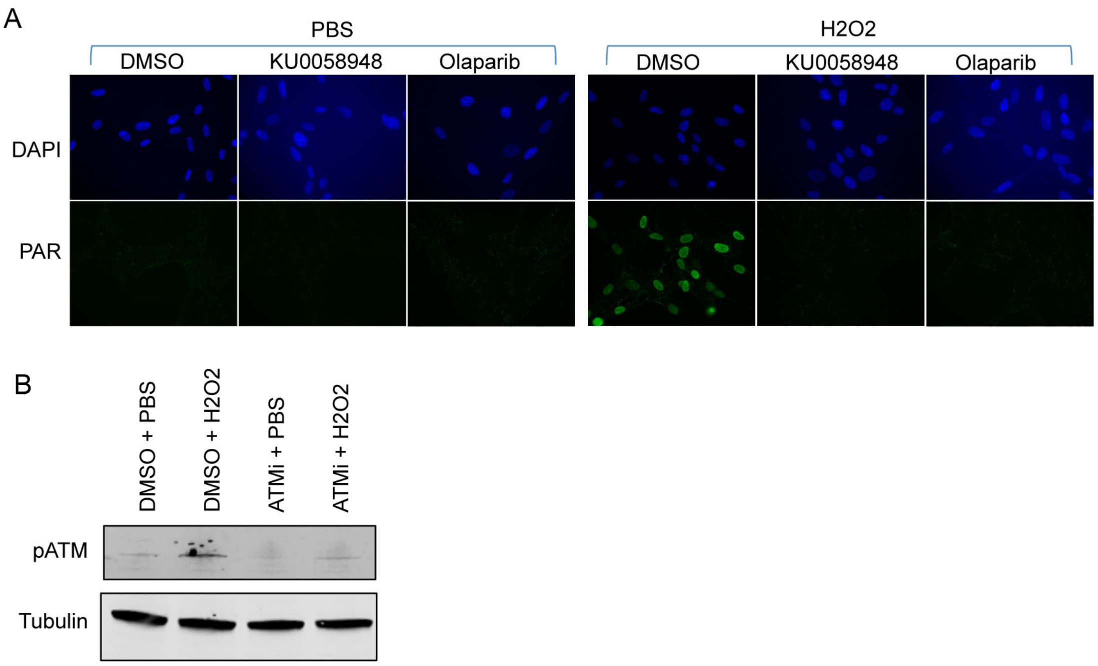

Supplement: Supplementary file 7 — Additional file 7: Figure S7. Validation of inhibitor activity. [file 12915_2020_771_MOESM7_ESM.pdf]
