# Supplementary material for: Persistent DNA damage triggers activation of the integrated stress response to promote cell survival under nutrient restriction
Source: BMC Biol. 2020 Mar 30;18:36. doi: 10.1186/s12915-020-00771-x (PMC7106853; doi:10.1186/s12915-020-00771-x)

Clementi *et al,* 2020

Additional Figure S8: No influence of XRCC1 KD in siSp1 or IR treated cells grown at 5% FCS. A-B) Comparable growth of siCon and siSp1 cells at 5% FCS. Phase-contrast images of Control (A) or Sp1 KD (B) cells. Images are from one representative experiment (from a total of n = 3 independent experiments), with two different fields randomly chosen on each plate shown per condition. Scale bar = 400 µm. C-F) Growth of control or IR treated cells at 5% FCS. Phase-contrast images of Control (C) or cells treated with different amounts of IR (D–F). Images are from one representative experiment (from a total of n = 4 independent experiments), with two different fields randomly chosen on each plate shown per condition.

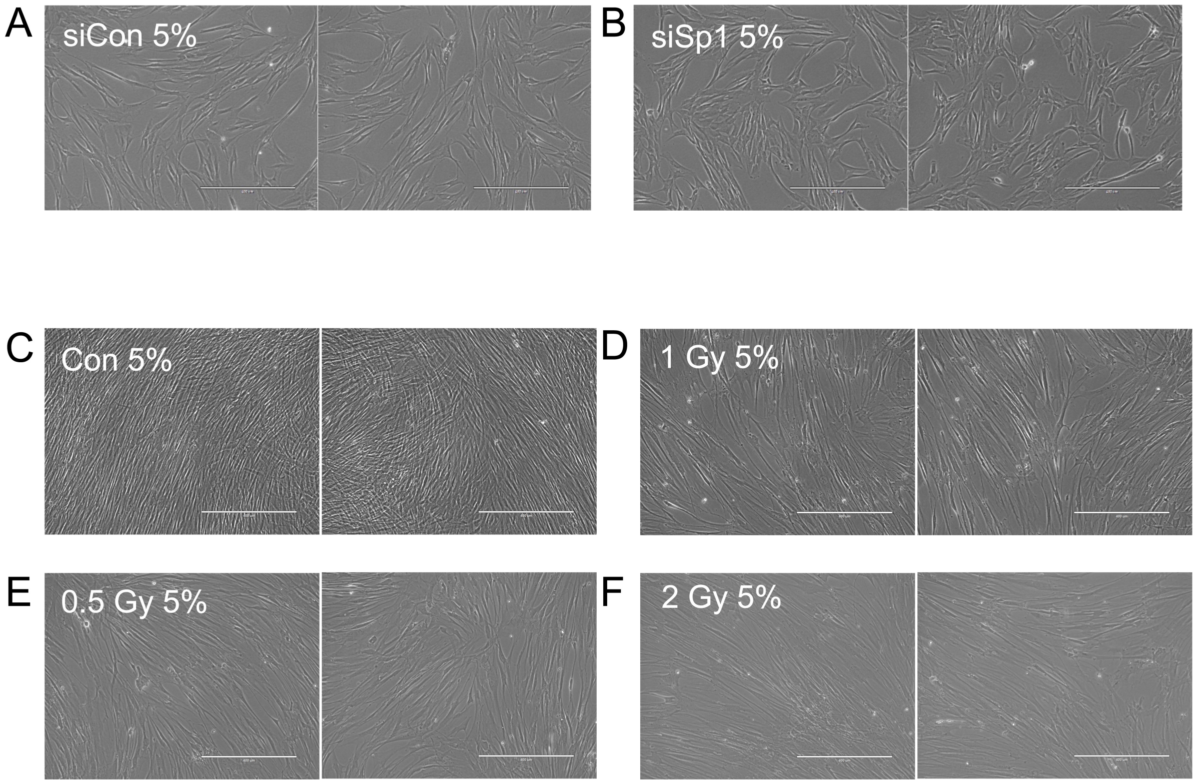

Supplement: Supplementary file 8 — Additional file 8: Figure S4. No influence of XRCC1 KD in siSp1 or IR treated cells grown at 5% FCS. [file 12915_2020_771_MOESM8_ESM.pdf]
