# Supplementary material for: Persistent DNA damage triggers activation of the integrated stress response to promote cell survival under nutrient restriction
Source: BMC Biol. 2020 Mar 30;18:36. doi: 10.1186/s12915-020-00771-x (PMC7106853; doi:10.1186/s12915-020-00771-x)
Supplement: Supplementary file 9 — Additional file 9: Table S1. siRNA sequences used in this study. [file 12915_2020_771_MOESM9_ESM.pdf]

Additional Table S1: siRNA sequences used in this study. *ATF4 siRNA sequences were used in combination as a pool.

**siRNA**	**Sequence**	**Reference/order no.**	**Manufacturer**
siControl	Manufacturer's proprietary information	SR-CL000-005	Eurogentec
siXRCC1#1	5'-AGGGAAGAGGAAGUUGGAU-3'	[28]	Eurogentec
siXRCC1#2	5'-GCUUGAGUUUUGUACGGUU-3'	[28]	Eurogentec
siXRCC1#3	5'-GCUUGAGUUUUGUACGGUU-3'	[39, 26]	Eurogentec
siATF4-1*	5'-GCCUAGGUCUCUUAGAUGA-3'	[28]	Eurogentec
siATF4-2*	5'-CUGCUUACGUUGCCAUGAU-3'	[28]	Eurogentec
siGCN2	5'-GGUGAAGAAGUAUAUGUAATT-3'	siRNA-ID s532694	ThermoFisher
siPERK	5'-GUGACGAAAUGGAACAAGA-3'	Assay ID s18102	ThermoFisher
