# Supplementary material for: Persistent DNA damage triggers activation of the integrated stress response to promote cell survival under nutrient restriction
Source: BMC Biol. 2020 Mar 30;18:36. doi: 10.1186/s12915-020-00771-x (PMC7106853; doi:10.1186/s12915-020-00771-x)
Supplement: Supplementary file 10 — Additional file 10: Table S2. List of primers used for qRT-PCR. [file 12915_2020_771_MOESM10_ESM.pdf]

Clementi *et al,* 2020

Additional Table S1: List of primers used for qRT-PCR.

Gene target	Sequence	Reference
XRCC1	Fw: 5’-AACACGGACAGTGAGGAACA-3’<br>Re: 5’-GCTGTGACGTATCGGATGAG-3’	[28]
ATF4	Fw: 5’-GGGACAGATTGGATGTTGGAGA -3’<br>Re: 5’-ACCCAACAGGGCATCCAAGT-3’	[28]
ACTA2	Fw: 5’-TCAATGTCCCAGCCATGTAT-3’<br>Re: 5’-CAGCACGATGCCAGTTGT-3’	[56]
PALLD	Fw: 5’-AACCGAGCAGGACAGAAC-3’<br>Re: 5’-TGGTGGCACTCCCAATAC-3’	[26]
PSAT1	Fw: 5’-CGGTCCTGGAATACAAGGTG-3’<br>Re: 5’-AACCAAGCCCATGACGTAGA-3’	[28]
B2M	Fw: 5’-ATGTCTCGCTCCGTGGCCTTA-3’<br>Re: 5’-ATCTTGGGCTGTGACAAAGTC -3’	[26]
GAPDH	Fw: 5’-AGCCACATCGCTCAGACAC-3’<br>Re: 5’-GCCCAATACGACCAAATCC-3’	[28]
