# Supplementary material for: Persistent DNA damage triggers activation of the integrated stress response to promote cell survival under nutrient restriction
Source: BMC Biol. 2020 Mar 30;18:36. doi: 10.1186/s12915-020-00771-x (PMC7106853; doi:10.1186/s12915-020-00771-x)
Supplement: Supplementary file 11 — Additional file 11: Table S3. List of primary antibodies used. [file 12915_2020_771_MOESM11_ESM.pdf]

Clementi *et al,* 2020

Additional Table S1: List of primary antibodies used.

Target	Manufacturer	Order no.	Batch no.	RRID
PALLD	Novus Biologicals	NBP1-25959	A-6	AB_1726016
PSAT1	Sigma	HPA042924	R40839	AB_2678223
αSMA	Dako Cytomation	M0851	00028353	AB_2223500
GAPDH	Abcam	ab9484	n.a.	AB_307274
α-Tubulin	Sigma	T5168	n.a.	AB_477579
XRCC1	Neomarkers	MS-1393-P1	1393P1512A	AB_62805
ATF4	Abcam	ab184909	GR3212613-9	AB_2819059
p-eIF2α	Abcam	ab32157	GR319444-17	AB_732117
PERK	Cell signalling	#3192	9	AB_2095847
ATM	Cell signalling	#2873	3	AB_2062659
p-ATM	Abcam	ab81292	n.a.	AB_1640207
PAR	Trevigen	4336-BPC-100	38194M16	AB_2721257
Sp1	Millipore	07-645	2884551	AB_310773

n.a. = information not available
